# Supplementary material for: Nitrogen fixation under declining Arctic sea ice
Source: Commun Earth Environ. 2025 Oct 20;6(1):811. doi: 10.1038/s43247-025-02782-4 (PMC12537486; doi:10.1038/s43247-025-02782-4)
Supplement: Supplementary file 1 — Supplementary material [file 43247_2025_2782_MOESM1_ESM.pdf]

Supplementary figures and tables

Supplementary figures S1-S9 and tables S1-S3 to the article:

“Nitrogen fixation under declining Arctic sea ice”

Lisa W. von Friesen, Hanna Farnelid, Wilken J. von Appen, Mar Benavides, Olivier Grosso, Christien P. Laber, Johanna Schüttler, Marcus Sundbom, Sinhué Torres-Valdés, Stefan Bertilsson, Ilka Peeken, Pauline Snoeijs-Leijonmalm, Lasse Riemann*

*Corresponding author: lriemann@bio.ku.dk

In addition to the figures and tables in the current file, the following supplementary data files are available at https://doi.org/10.6084/m9.figshare.29930714

Supplementary Datasheet 1. Rate and error-propagation calculations for nitrogen fixation rates and biomass-independent specific N_2-uptake.

Supplementary Datasheet 2. Rate calculations for carbon fixation rates.

Supplementary Datasheet 3. Data from the quantitative PCR of *nifH* from three non-cyanobacterial diazotroph assays: Beta-Arctic1, Gamma-Arctic1, and Gamma-Arctic2.

Nucleotide and amino acid lists of generated *nifH* amplicon sequence variants.

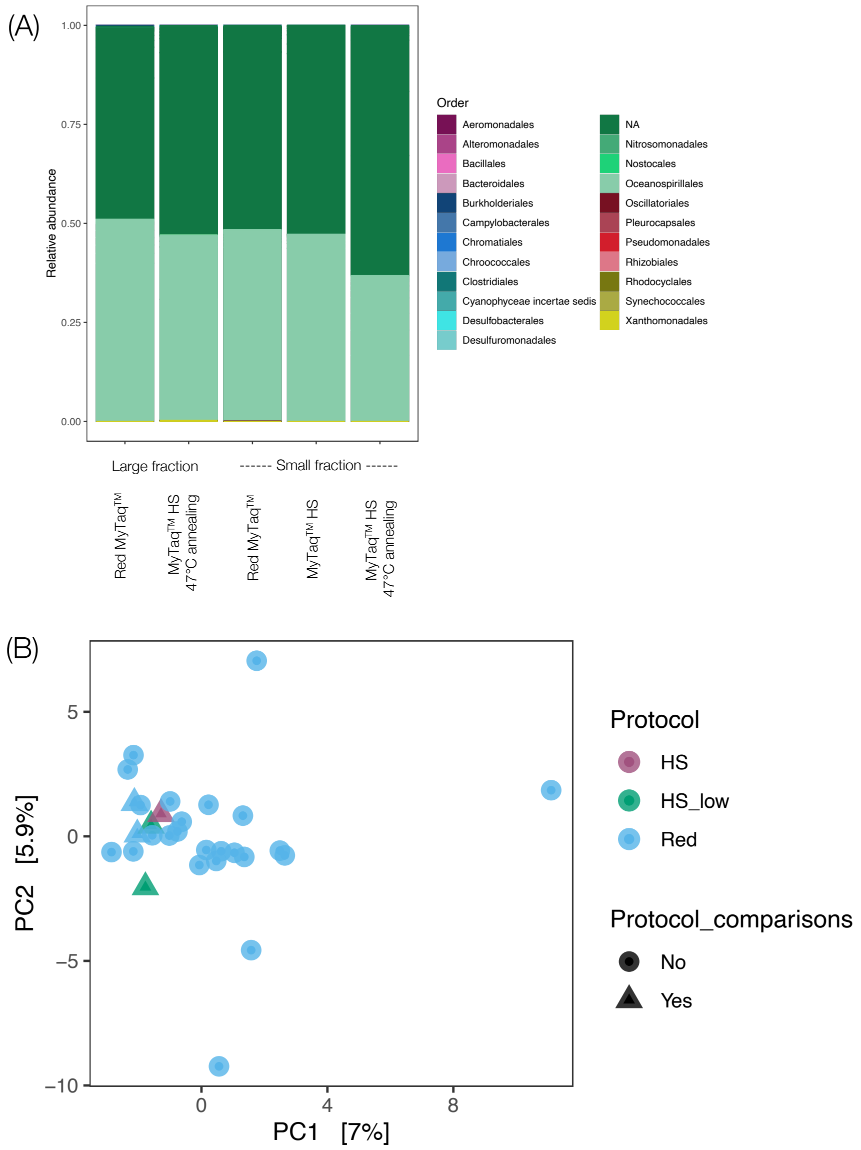

Figure S1. **PCR amplification protocol comparisons.** Comparison of *nifH* PCR amplification results from the three PCR protocols applied (HS: MyTaq™ HS, HS_low: MyTaq™ HS 47°C annealing temperature, Red: Red MyTaq™) on two samples (large and small size fraction from Station 26). A) Relative abundance of diazotroph orders between the three protocols for the two samples, and B) the two first principal components of principal component analysis (PCA) of all DNA samples from the Synoptic Arctic Survey (SAS) expedition 2021 (eigenvalues PC1: 96.3, PC2: 81.0), displaying the similarity of different PCR protocols. NA: not assigned.

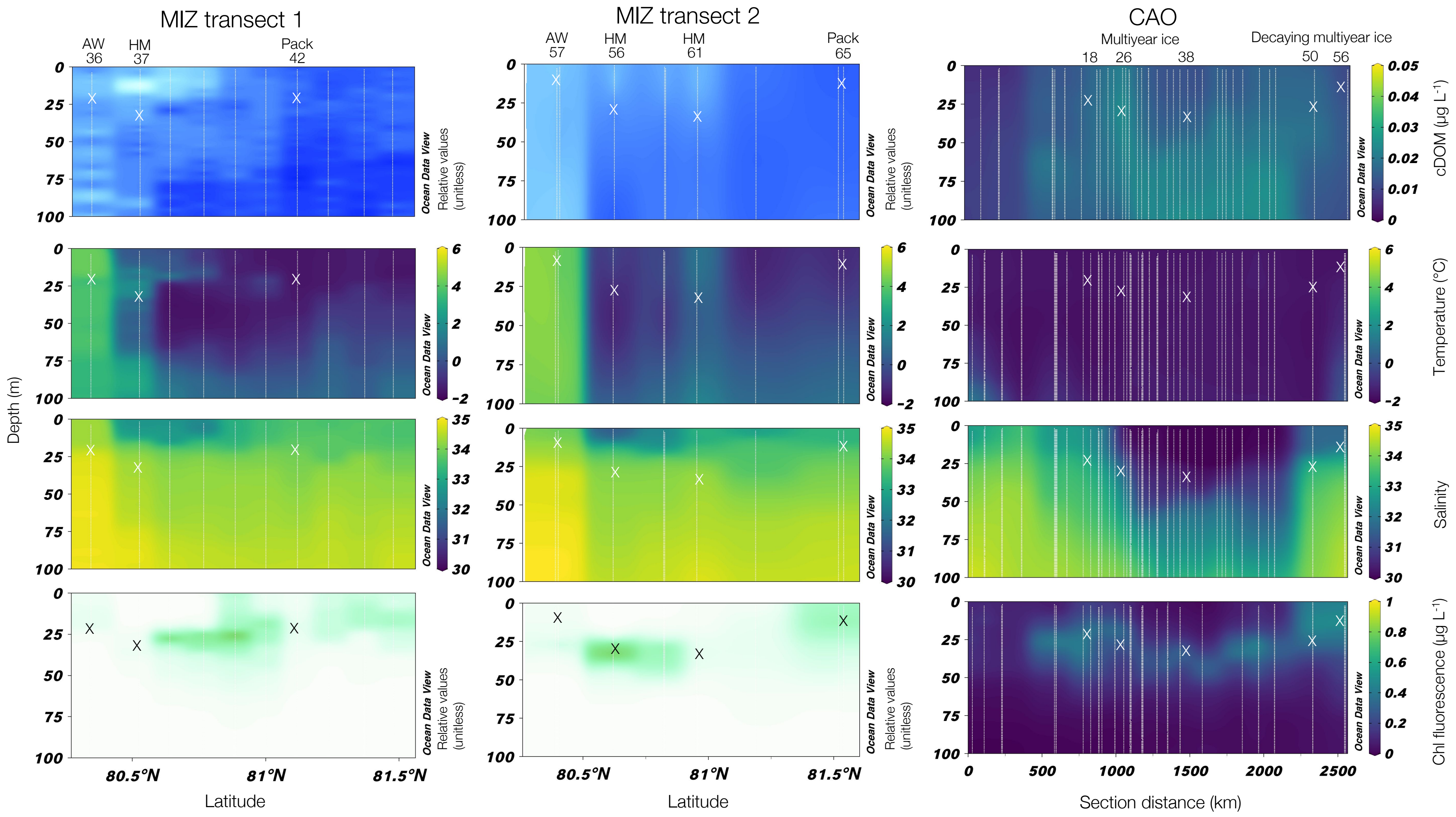

The figure legend is on the next page.

Figure S2. **Hydrographical conditions.** Hydrographical conditions of the top 100 m of the water column over the marginal ice zone (MIZ) transect one and two and the Central Arctic Ocean (CAO) cruise tracks as derived from CTD profiles (white vertical lines). White or black “X” denotes sampling depth, targeting the deep chlorophyll a maximum (DCM). Weighted-average gridding with a scale length of 65 permille was applied. cDOM: coloured dissolved organic matter, Chl fluorescence: chlorophyll a fluorescence. Note that no scaled colour bars are provided for cDOM and the Chl fluorescence from the MIZ transects due to raw relative values (i.e., no absolute values; darker nuance is relatively higher than lighter). AW: Atlantic open water, HM: high melt (pulse of meltwater), Pack: pack ice. Visualised with Ocean Data View (v.5.6.2; Schlitzer 2022).

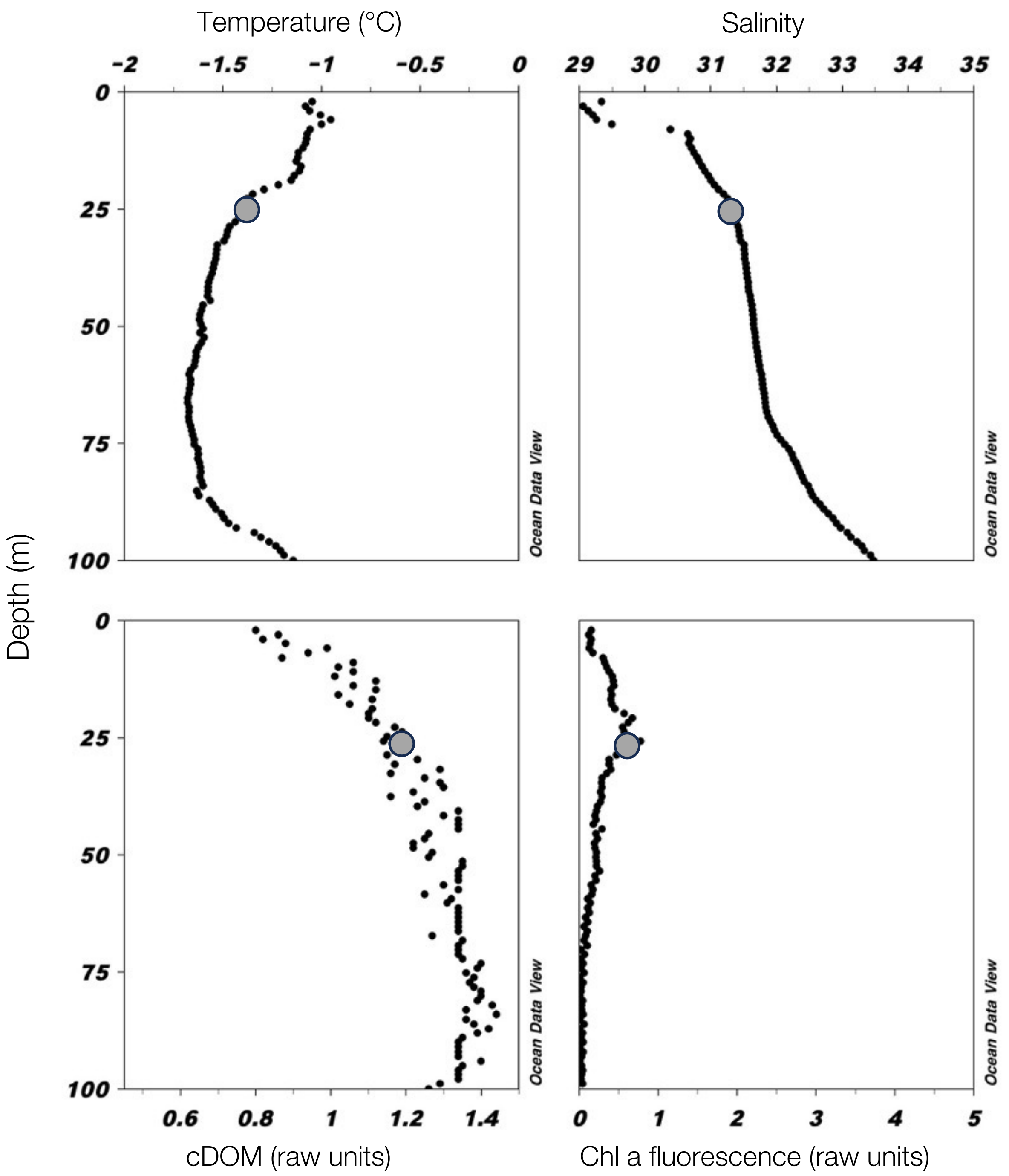

Figure S3. **Hydrographical conditions at Station 108.** Hydrographical conditions of the top 100 m of the water column at Station 108 (land-fast ice, northeast Kalaallit Nunaat (Greenland)). The grey circle denotes the sampling depth (deep chlorophyll a maximum of 25 m). cDOM: coloured dissolved organic matter. Chl a: chlorophyll a.

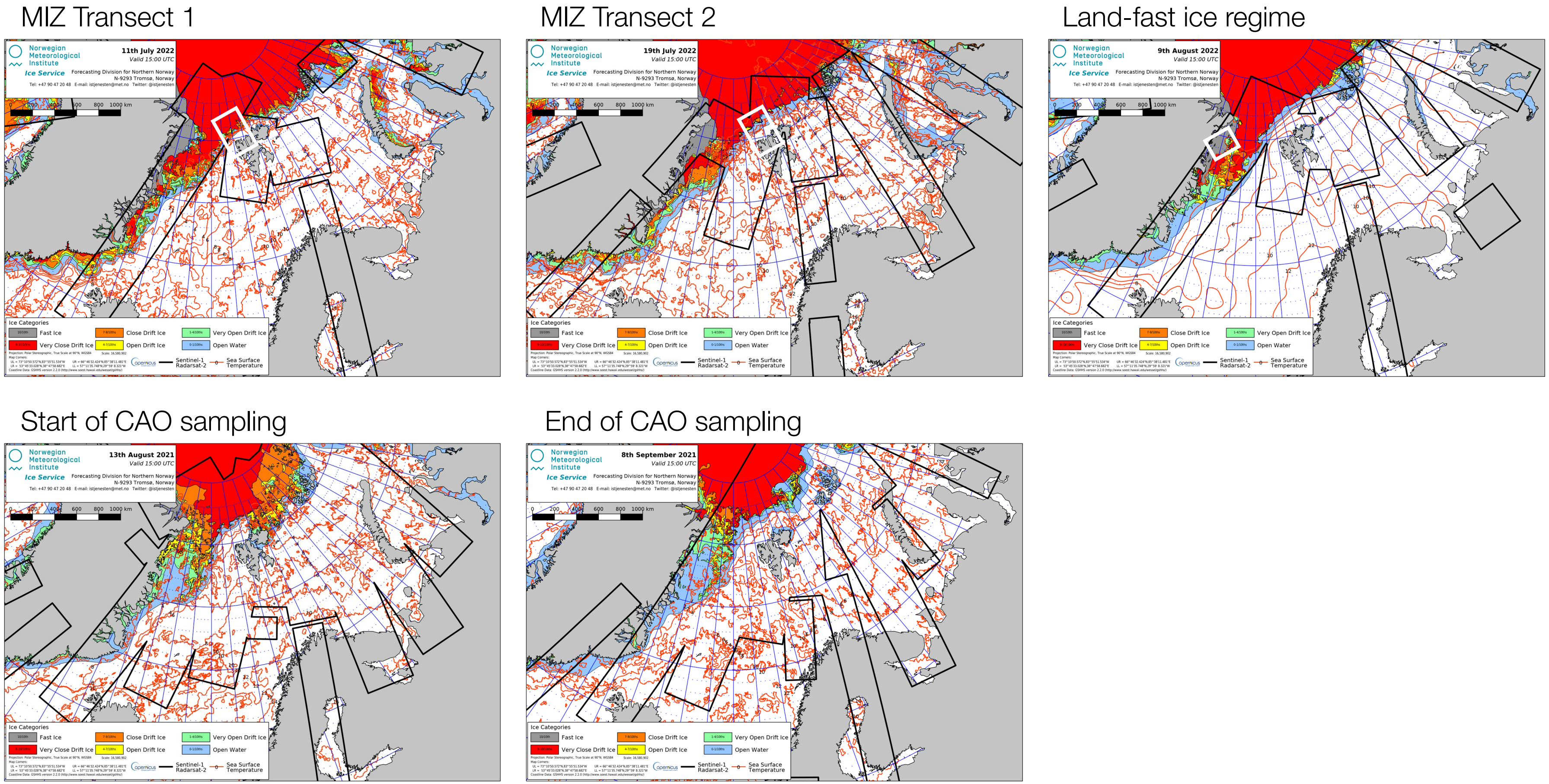

Figure S4. **Sea ice conditions during the sampling periods.** Sea ice maps are obtained from the Ice Service of the Norwegian Meteorological Institute (MET, http://polarview.met.no/) for relevant dates of the Central Arctic Ocean (CAO), marginal ice zone (MIZ) transect one and transect two samplings. White boxes denote sampling regions.

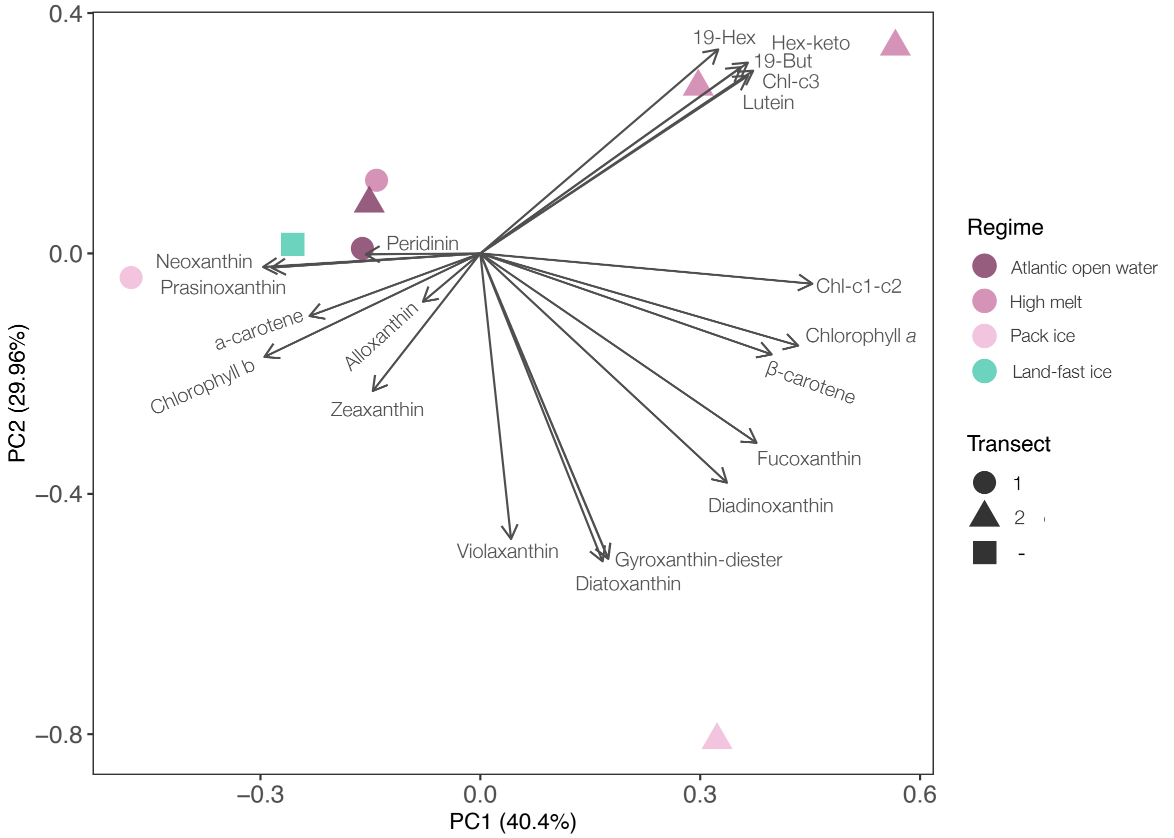

Figure S5. **Pigment composition of samples from the ATWAICE cruise 2022**. The two first principal components of principal component analysis (PCA) of the pigment composition at the deep chlorophyll a maximum (eigenvalues PC1: 8.48, PC2: 6.29). Transects one and two refer to the transects performed over the MIZ; the square symbol displays Station 108 (land-fast ice, northeast Greenland). See Table S3 for absolute values and explanations of abbreviations.

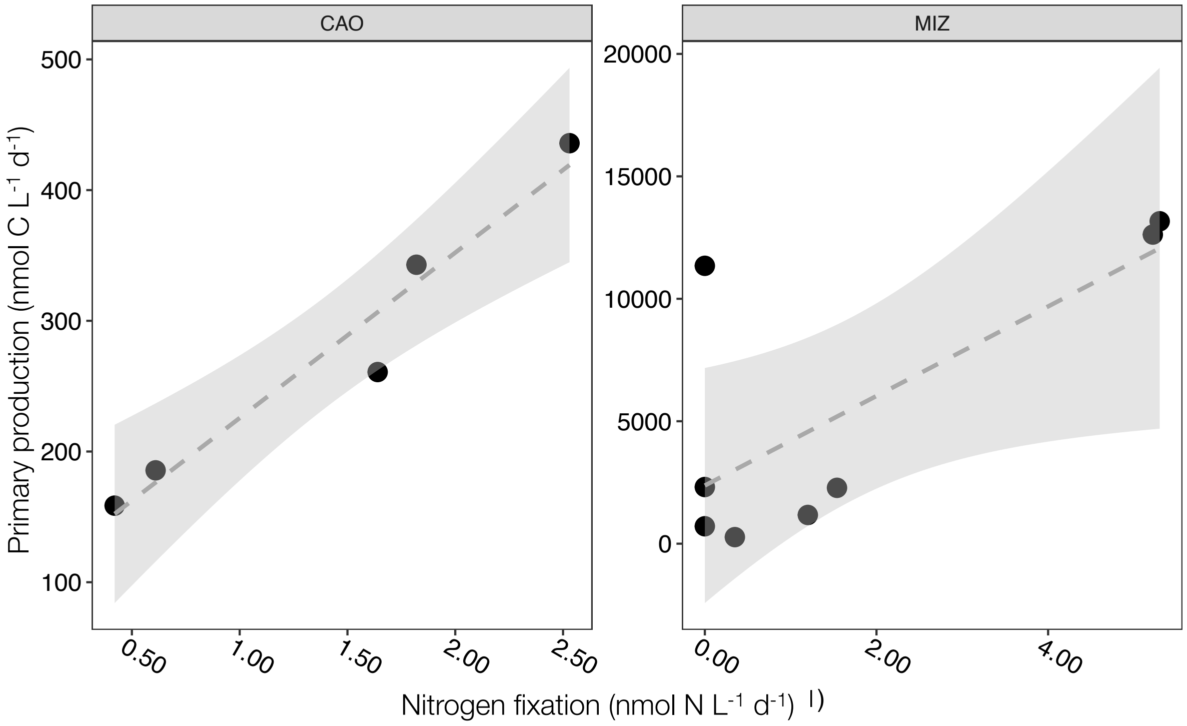

Figure S6. **Relationship between nitrogen fixation and primary production**. LM (linear model) smoothing with a confidence interval of 95%. CAO: Central Arctic Ocean, MIZ: Marginal Ice Zone.

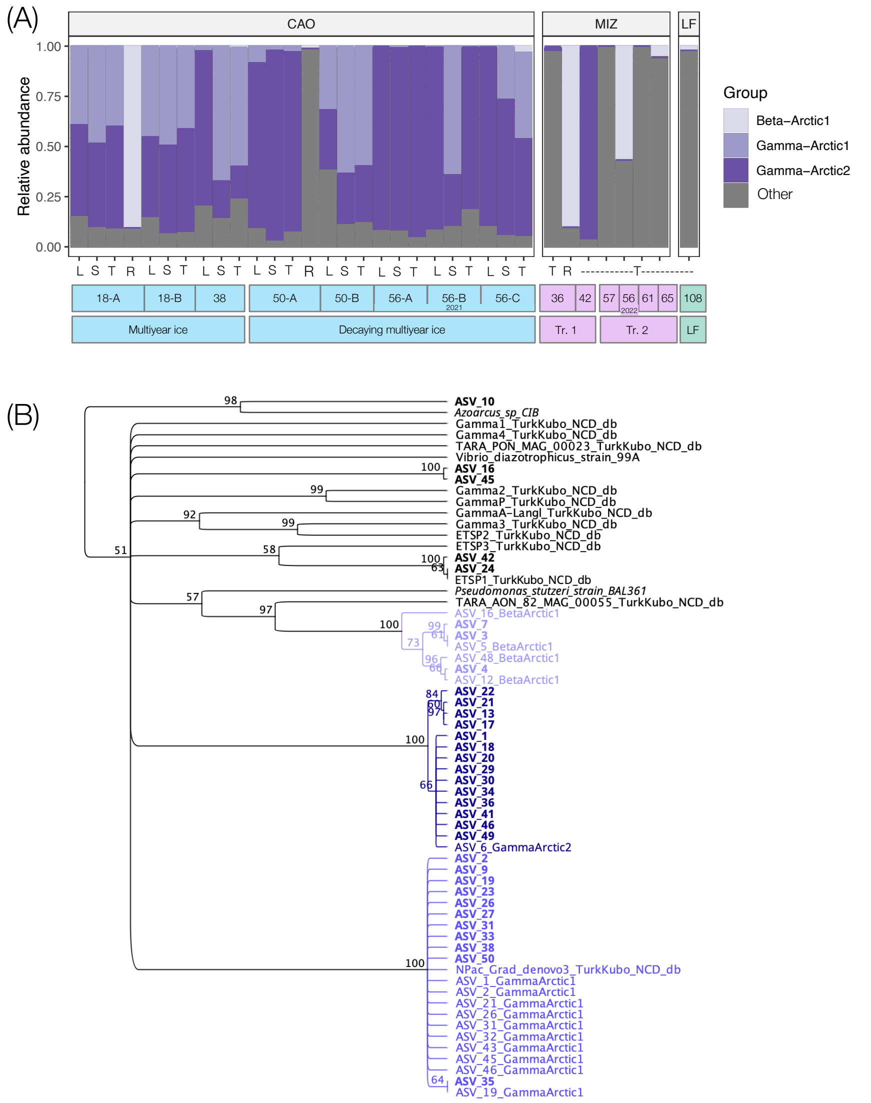

Figure S7. **Arctic key non-cyanobacterial diazotrophs.** A) Relative abundance and *nifH* gene expression of non-cyanobacterial diazotrophs proposed as Arctic key groups (Beta-Arctic1, Gamma-Arctic1, Gamma-Arctic2; von Friesen et al. 2025) at each station. CAO: Central Arctic Ocean, MIZ: marginal ice zone, LF: land-fast ice. L: large size fraction (>2 µm), S: small size fraction (0.22 µm), T: total (>0.22 µm) size fractions (equal volume of DNA template from >2 and 0.22 µm size fractions for comparability of CAO to MIZ and LF, see methods), R: RNA (*nifH* expression). Note one to three replicates (A-C) for each station in the CAO. Tr. 1: transect one, Tr. two: transect 2, “Other”: all other groups. B) UPGMA phylogenetic tree of partial *nifH* nucleotide sequences (mafft alignment, Jukes-Cantor distance model, 1000 bootstraps; GeneiousPrime v.2022.1.1). The phylogenetic relationship of the top 50 ASVs (in bold) assigned as Gamma- or Betaproteobacteria to the three previously proposed key NCDs Beta-Arctic1, Gamma-Arctic1 or Gamma-Arctic2 (von Friesen et al. 2025). Additional reference sequences labelled as “TurkKubo_NCD_db” are from a recently compiled NCD database (Turk-Kubo et al., 2022).

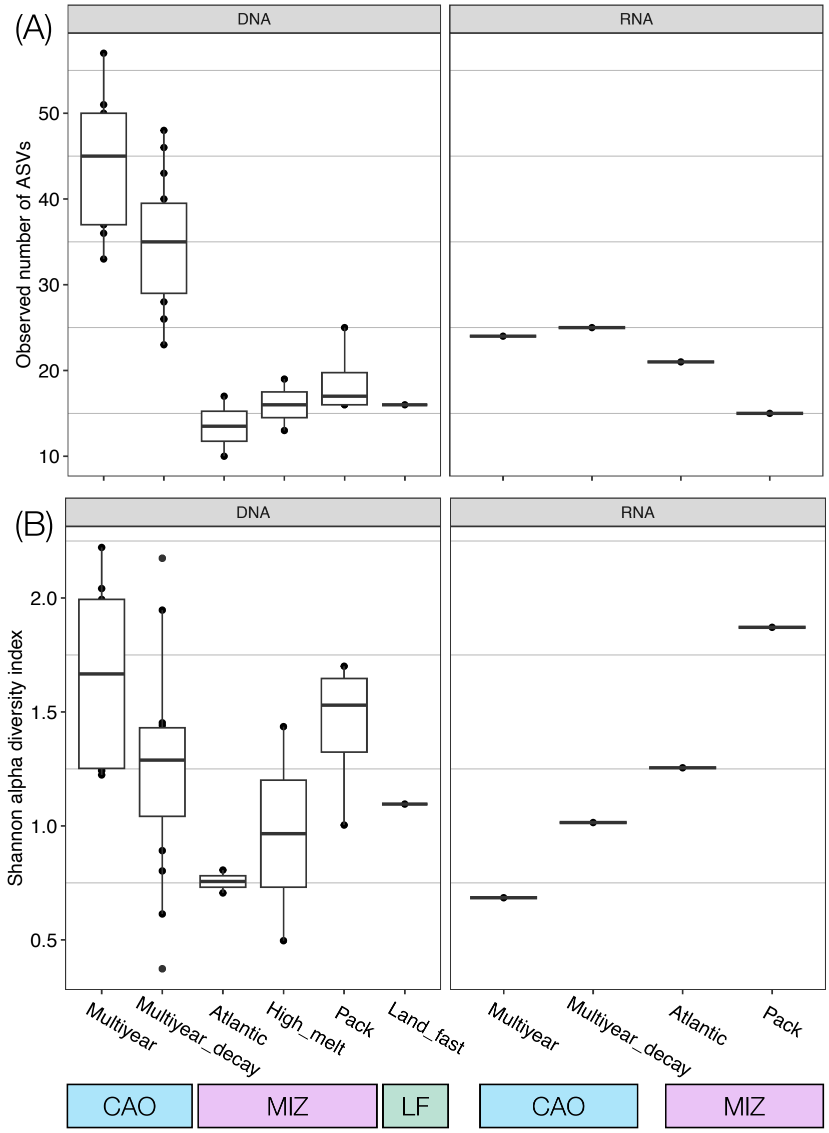

Figure S8. **Diazotroph alpha diversity in different regimes.** (A) The number of observed *nifH* amplicon sequence variants (ASVs) and (B) Shannon alpha diversity index in each sea ice regime for DNA and RNA separately. CAO: Central Arctic Ocean, MIZ: marginal ice zone, LF: land-fast ice.

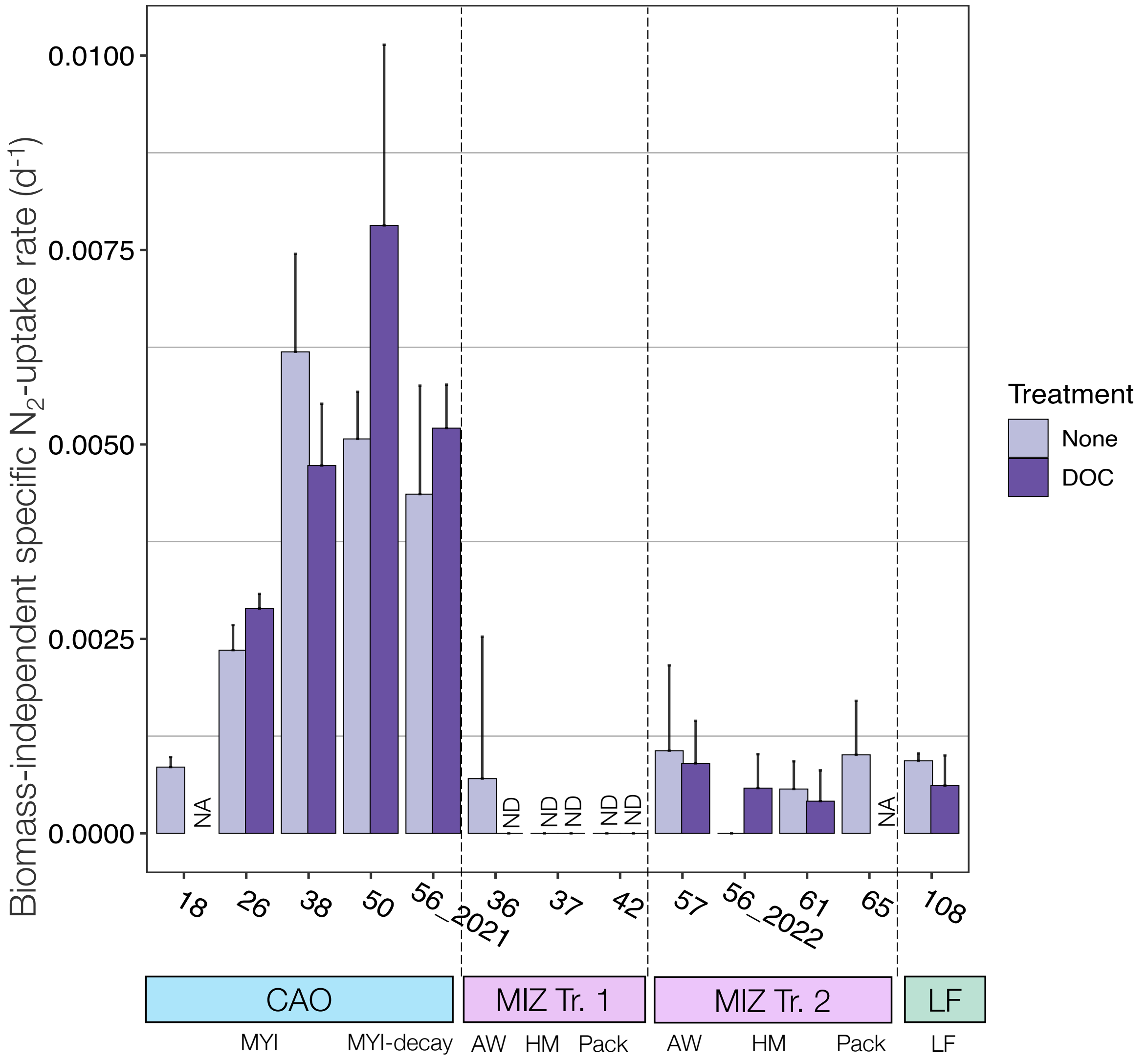

Figure S9. **Biomass independent specific N_2-uptake rates.** Specific N2-uptake rates at each station. Error bars represent one standard deviation. NA: not analysed, ND: not detected (below the limit of detection; Supplementary Datasheets S1, S2), DOC: dissolved organic carbon treatment (see methods), CAO: central Arctic Ocean, MIZ: marginal ice zone, Tr. 1: transect 1, Tr. 2: transect 2, MYI: multiyear ice, MYI-decay: decaying multiyear ice, AW: Atlantic open water, HM: high melt, Pack: pack ice, LF: land-fast sea ice.

Table S1. **Sampling information, coordinates, and conditions.** SAS: Synoptic Arctic Survey, CAO: Central Arctic Ocean, MIZ: marginal ice zone, LF: land-fast ice, DCM: deep chlorophyll *a* maximum, UTC: coordinated universal time. Coordinates are given in decimal degrees.

Station	Cruise	Region	Regime	Latitude (N)	Longitude (E)	Date	Sampling time (UTC)	DCM depth (m)	~Ice thickness (cm)	Weather
18	SAS	CAO	Multiyear ice	89.1755	22.7822	8/13/21	9:55:00 AM	22	160	Sunny
26	SAS	CAO	Multiyear ice	89.1381	-150.9231	8/20/21	5:58:00 AM	30	190	Cloudy
38	SAS	CAO	Multiyear ice	87.7780	-64.2439	8/29/21	9:50:00 AM	33	120	Cloudy/Sunny
50	SAS	CAO	Decaying multiyear ice	84.1517	-31.7415	9/4/21	7:14:00 PM	28	160	Cloudy
56_2021	SAS	CAO	Decaying multiyear ice	83.8326	-1.1049	9/8/21	9:00:00 PM	15	110	Cloudy
36	ATWAICE	MIZ	Atlantic open water	80.3455	10.6092	7/11/22	11:40:00 AM	20	-	Cloudy, moderate swell
37	ATWAICE	MIZ	High-melt zone	80.5265	10.1144	7/11/22	5:05:00 PM	35	<110	Cloudy
42	ATWAICE	MIZ	Pack ice (first year ice)	81.1187	8.2453	7/12/22	4:32:00 AM	20	130	Fog
56_2022	ATWAICE	MIZ	High-melt zone	80.6202	9.7851	7/17/22	4:54:00 PM	31	<110	Sunny, 2-3 m swell
57	ATWAICE	MIZ	Atlantic open water	80.3900	10.4719	7/18/22	12:26:00 AM	8	-	Cloudy
61	ATWAICE	MIZ	High-melt zone	80.8262	9.1396	7/18/22	9:58:00 PM	36	<110	Sunny
65	ATWAICE	MIZ	Pack ice (first year ice)	81.5391	7.1263	7/19/22	5:38:00 AM	11	130	Cloudy
108	ATWAICE	LF	Land-fast ice	78.6494	-17.5750	8/6/22	12:33:00 AM	25	<100	Cloudy

Table S2. **Primers and probes** used in this study for the amplification and quantification of *nifH* genes.

Primer name	Sequence (5'-3')	Type	Reference
nifH1	TGYGAYCCNAARGCNGA	Amplicon sequencing	Zehr & McReynolds, 1989
nifH2	ADNGCCATCATYTCNCC		
nifH3	ATRTTRTTNGCNGCRTA		Zani et al., 2000
nifH4	TTYTAYGGNAARGGNGG		
Gamma-Arctic1 forward	ATTATGGAAATGGCGGCAG	Quantitative PCR	von Friesen et al., 2025
Gamma-Arctic1 reverse	GACCACCAGATTCAACACAG		
Gamma-Arctic1 probe	CGGCACCGTAGAAGACCTTGAACTTGAA		
Gamma-Arctic2 forward	ATCATGGAGATGGCTG		
Gamma-Arctic2 reverse	GACCACCGGACTCAACACAC		
Gamma-Arctic2 probe	AGGTACTGTAGAAGATCTGGAACTGGAA		
Beta-Arctic1 forward	ATCAYCGCCATCAACTTCC		
Beta-Arctic1 reverse	ACGATGTAGATTTCCTGAGCC		
Beta-Arctic1 probe	CGGTGGCTTCGCCATGCCGAT		

Table S3. **Pigment concentrations during the ATWAICE campaign (2022).** The unit is μg L^{-1} for all pigments.

		Station							
Abbreviation	Full name	36	37	42	56_2022	57	61	65	108
Chl_a	Chlorophyll *a*	1.740	1.237	0.972	8.996	0.623	4.836	8.827	0.296
Chl_c3	Chlorophyll c3	0.271	0.334	0.044	2.289	0.085	1.376	0.106	0.019
Chl_c1_2	Chlorophyll c1+c2	0.311	0.233	0.109	1.833	0.090	1.129	1.407	0.039
Peridinin	Peridinin	0.025	0.000	0.007	0.000	0.000	0.000	0.000	0.000
Phorbid_a	Pheophorbide *a*	0.000	0.000	0.000	0.000	0.000	0.000	0.573	0.000
19_But	19′-butanoyloxyfucoxanthin	0.097	0.000	0.000	0.357	0.012	0.258	0.000	0.000
Fuco	Fucoxanthin	0.551	0.542	0.257	3.325	0.125	1.944	5.710	0.063
Neo	Neoxanthin	0.000	0.000	0.052	0.000	0.000	0.000	0.000	0.014
19_Hex	19′-hexanoyloxyfucoxanthin	0.626	0.141	0.000	1.202	0.268	0.993	0.000	0.000
Hex_keto	19'-hex-4-ketofucoxanthin	0.034	0.059	0.000	0.406	0.000	0.268	0.000	0.000
Prasino	Prasinoxanthin	0.000	0.000	0.135	0.000	0.000	0.000	0.000	0.024
Viola	Violaxanthin	0.012	0.000	0.000	0.000	0.006	0.000	0.026	0.014
Diadino	Diadinoxanthin	0.100	0.059	0.015	0.314	0.072	0.223	0.766	0.005
Allo	Alloxanthin	0.043	0.000	0.003	0.000	0.000	0.000	0.007	0.000
Diato	Diatoxanthin	0.005	0.000	0.000	0.000	0.000	0.000	0.051	0.000
Zea	Zeaxanthin	0.005	0.000	0.003	0.000	0.000	0.000	0.003	0.000
Lutein	Lutein	0.018	0.004	0.006	0.099	0.006	0.059	0.005	0.000
Gyro_diester	Gyroxanthin-diester	0.000	0.000	0.000	0.000	0.000	0.000	0.039	0.000
Chl_b	Chlorophyll *b*	0.068	0.036	0.455	0.000	0.027	0.000	0.134	0.153
a_carotene	*a* -carotene	0.000	0.000	0.034	0.000	0.000	0.000	0.005	0.000
ß_carotene	*ß* -carotene	0.000	0.000	0.036	0.115	0.025	0.089	0.129	0.009

Supplementary references

Turk-Kubo, K. A., M. R. Gradoville, S. Cheung, F. M. Cornejo-Castillo, K. J. Harding, M. Morando, M. Mills, and J. P. Zehr. 2022. Non-cyanobacterial diazotrophs: global diversity, distribution, ecophysiology, and activity in marine waters. FEMS Microbiol. Rev. 1–25. doi:10.1093/femsre/fuac046

von Friesen, L. W., C. P. Laber, B. H. Kristensen, and others. 2025. From temperate to polar waters: Transition to non-cyanobacterial diazotrophy upon entering the Atlantic gateway of the Arctic Ocean. bioRxiv. doi:10.1101/2025.01.27.635040

Schlitzer, R. 2022. Ocean Data View. https://odv.awi.de

Zani, S., M. T. Mellon, J. L. Collier, and J. P. Zehr. 2000. Expression of *nifH* genes in natural microbial assemblages in Lake George, New York, detected by reverse transcriptase PCR. Appl. Environ. Microbiol. 66: 3119–3124. doi:10.1128/AEM.66.7.3119-3124.2000

Zehr, J. P., and L. A. McReynolds. 1989. Use of degenerate oligonucleotides for amplification of the *nifH* gene from the marine cyanobacterium *Trichodesmium thiebautii*. Appl. Environ. Microbiol. 55: 2522–2526. doi:10.1128/aem.55.10.2522-2526.1989
