# Supplementary material for: Nitrogen fixation under declining Arctic sea ice
Source: Commun Earth Environ. 2025 Oct 20;6(1):811. doi: 10.1038/s43247-025-02782-4 (PMC12537486; doi:10.1038/s43247-025-02782-4)
Supplement: Supplementary file 2 — Description of Additional Supplementary Materials [file 43247_2025_2782_MOESM2_ESM.pdf]

Description of Additional Supplementary Files

File name: Supplementary Datasheet 1

Description: Rate and error-propagation calculations for nitrogen fixation rates and specific N_2-uptake.

File name: Supplementary Datasheet 2

Description: Rate calculations for carbon fixation rates.

File name: Supplementary Datasheet 3

Description: Data from the quantitative PCR of *nifH* from three non-cyanobacterial diazotroph assays: Beta-Arctic1, Gamma-Arctic1, and Gamma-Arctic2.
