# Supplementary material for: Nitrogen fixation under declining Arctic sea ice
Source: Commun Earth Environ. 2025 Oct 20;6(1):811. doi: 10.1038/s43247-025-02782-4 (PMC12537486; doi:10.1038/s43247-025-02782-4)
Supplement: Supplementary file 6 — Reporting summary [file 43247_2025_2782_MOESM6_ESM.pdf]

nature portfolio

Corresponding author(s): Lasse Riemann

Last updated by author(s): Aug 18, 2025

Reporting Summary

Nature Portfolio wishes to improve the reproducibility of the work that we publish. This form provides structure for consistency and transparency in reporting. For further information on Nature Portfolio policies, see our Editorial Policies and the Editorial Policy Checklist.

Statistics

For all statistical analyses, confirm that the following items are present in the figure legend, table legend, main text, or Methods section.

n/a	Confirmed	
☐	☒	The exact sample size (*n*) for each experimental group/condition, given as a discrete number and unit of measurement
☐	☒	A statement on whether measurements were taken from distinct samples or whether the same sample was measured repeatedly
☐	☒	The statistical test(s) used AND whether they are one- or two-sided<br>*Only common tests should be described solely by name; describe more complex techniques in the Methods section.*
☐	☒	A description of all covariates tested
☐	☒	A description of any assumptions or corrections, such as tests of normality and adjustment for multiple comparisons
☐	☒	A full description of the statistical parameters including central tendency (e.g. means) or other basic estimates (e.g. regression coefficient) AND variation (e.g. standard deviation) or associated estimates of uncertainty (e.g. confidence intervals)
☐	☒	For null hypothesis testing, the test statistic (e.g. *F*, *t*, *r*) with confidence intervals, effect sizes, degrees of freedom and *P* value noted<br>*Give P values as exact values whenever suitable.*
☒	☐	For Bayesian analysis, information on the choice of priors and Markov chain Monte Carlo settings
☒	☐	For hierarchical and complex designs, identification of the appropriate level for tests and full reporting of outcomes
☒	☐	Estimates of effect sizes (e.g. Cohen's *d*, Pearson's *r*), indicating how they were calculated

Our web collection on statistics for biologists contains articles on many of the points above.

Software and code

Policy information about availability of computer code

Data collection	Statistical analyses were performed in R (v.4.1.0), and data were visualised with ggplot2 (v.3.4.0; Wickham 2016), ggOceanMaps (v.1.3.4; Vihtakari 2022), and Ocean Data View (v.5.6.2; Schlitzer 2022).
Data analysis	The following R-packages were used for data analysis: phyloseq (v.1.36.0; McMurdie and Holmes 2013), vegan (v.2.6-2; Oksanen et al. 2022), microViz (v.0.10.0; Barnett et al. 2021), PairwiseAdonis (v.0.4; Martinez Arbizu 2020), decontam (v.1.12.0; Davis et al. 2018). The following code/pipeline/software was used in data analysis: DADA2 (v.1.20.0; Callahan et al. 2016), parts of the NifMAP pipeline to exclude potential nifH homologs (v.1.0; Angel et al. 2018), Classification and Regression Trees CART for nifH phylogenetic cluster assignment (Frank et al., 2016), nifH database (Moynihan 2020),

For manuscripts utilizing custom algorithms or software that are central to the research but not yet described in published literature, software must be made available to editors and reviewers. We strongly encourage code deposition in a community repository (e.g. GitHub). See the Nature Portfolio guidelines for submitting code & software for further information.

Data

Policy information about availability of data

All manuscripts must include a data availability statement. This statement should provide the following information, where applicable:

- Accession codes, unique identifiers, or web links for publicly available datasets
- A description of any restrictions on data availability
- For clinical datasets or third party data, please ensure that the statement adheres to our policy

The data that supports the findings of this study are available in the supplementary material of this article, openly available in PANGAEA at https://doi.org/10.1594/PANGAEA.951266 and https://doi.org/10.1594/PANGAEA.956136, and in the National Centre for Biotechnology Information (NCBI) Sequence Read Archive (SRA), reference number PRJNA995422. Supplementary data files are available at https://doi.org/10.6084/m9.figshare.29930714.

Research involving human participants, their data, or biological material

Policy information about studies with human participants or human data. See also policy information about sex, gender (identity/presentation), and sexual orientation and race, ethnicity and racism.

Reporting on sex and gender	Not applicable.
Reporting on race, ethnicity, or other socially relevant groupings	Not applicable.
Population characteristics	Not applicable.
Recruitment	Not applicable.
Ethics oversight	Not applicable.

Note that full information on the approval of the study protocol must also be provided in the manuscript.

Field-specific reporting

Please select the one below that is the best fit for your research. If you are not sure, read the appropriate sections before making your selection.

☐ Life sciences ☐ Behavioural & social sciences ☒ Ecological, evolutionary & environmental sciences

For a reference copy of the document with all sections, see nature.com/documents/nr-reporting-summary-flat.pdf

Ecological, evolutionary & environmental sciences study design

All studies must disclose on these points even when the disclosure is negative.

Study description	The study investigates nitrogen fixation and diazotrophs at the deep chlorophyll a maximum in the Arctic Ocean and is based on two scientific cruises in different regions of the Arctic Ocean. The first cruise was conducted with IB Oden during the Swedish Synoptic Arctic Survey (SAS) expedition from 26 July to 19 September 2021. The second cruise was conducted with RV Polarstern during the PS131 expedition (ATWAICE) from 28 June to 17 August 2022 at the Yermak Plateau north of Svalbard, where a two-times repeated transect for the assessment of temporal patterns across various sea ice regimes (from Atlantic open water to first-year pack ice) in the marginal ice zone (MIZ) was performed. Experimental amendment of dissolved organic carbon mixture was performed (n=3) in parallel to the unmodified incubations (n=3) at each station.
Research sample	The research sample is Arctic Ocean seawater and it's microorganisms. The seawater was prefiltered through 200 um mesh to remove larger grazers that could unproportionally interfere with the incubation condition in individual bottles.
Sampling strategy	Sampling was conducted with a CTD/Rosette sampler from the target depth. Water was collected from niskin bottles into acid washed polycarbonate bottles. The incubated volume was based on expected POC/PN concentrations and the known limit of detection of the EA-IRMS instrument. A larger volume was chosen due to the expected low biomass conditions of the Arctic Ocean, and to decrease the bottle effect.
Data collection	Took place from 26 July to 19 September 2021 and 28 June to 17 August 2022. Lisa von Friesen was the main responsible person for sample collection and documentation during the field work.
Timing and spatial scale	Cruise 2 was performed as two transects across the marginal ice zone and in land-fast sea ice cover close to the Greenland coast. Cruise 1 was performed as one continuous cruise track through the central Arctic Ocean.
Data exclusions	No data was excluded.

Reproducibility	Repeated sampling was approached mainly during transect 2, but as the biological system in study changes on short time scales (e.g. ongoing bloom), it is not possible or ecologically relevant to re-visit the same coordinate and expect the same result. Rather, we describe the different conditions between the sampling occasions and utilize it to explain the observed patterns.
Randomization	Not applicable.
Blinding	Sample IDs for all parameters were set as a continuous number instead of a descriptor. This ensured blindness during the analyses of the different sample parameters (i.e. the person running a certain instrument did not know what station or treatment the current sample belonged to).

Did the study involve field work? ☒ Yes ☐ No

Field work, collection and transport

Field conditions	Field work took place during in total 3.5 months in two different years. Conditions are described in Table S1 and Figure 2.
Location	Arctic Ocean. All coordinates and depths are found in Table S1.
Access & import/export	Sampling collection took place with appropriate permits and customs handling from the cruise organizers (Alfred Wegener Institute, Germany, PS131 cruise 2022) (Swedish Polar Research Secretariat, Sweden, SAS-Oden2021 cruise 2021). Further information can be found in the cruise reports https://doi.org/10.57738/BzPM_0770_2023 and https://su.diva-portal.org/smash/record.jsf?pid=diva2%3A1729240&dswid=6888
Disturbance	All rules on minimizing environmental disturbance during sampling were followed according to the cruise organisers.

Reporting for specific materials, systems and methods

We require information from authors about some types of materials, experimental systems and methods used in many studies. Here, indicate whether each material, system or method listed is relevant to your study. If you are not sure if a list item applies to your research, read the appropriate section before selecting a response.

Materials & experimental systems

n/a	Involved in the study
☒	☐ Antibodies
☒	☐ Eukaryotic cell lines
☒	☐ Palaeontology and archaeology
☒	☐ Animals and other organisms
☒	☐ Clinical data
☒	☐ Dual use research of concern
☒	☐ Plants

Methods

n/a	Involved in the study
☒	☐ ChIP-seq
☐	☒ Flow cytometry
☒	☐ MRI-based neuroimaging

Plants

Seed stocks	Not applicable.
Novel plant genotypes	Not applicable.
Authentication	Not applicable.

Flow Cytometry

Plots

Confirm that:

- [ ] The axis labels state the marker and fluorochrome used (e.g. CD4-FITC).
- [ ] The axis scales are clearly visible. Include numbers along axes only for bottom left plot of group (a 'group' is an analysis of identical markers).
- [ ] All plots are contour plots with outliers or pseudocolor plots.
- [x] A numerical value for number of cells or percentage (with statistics) is provided.

Methodology

Sample preparation	Seawater for cell enumeration using flow cytometry (CYTOFlex, Beckman Coulter Inc, CA, USA; blue laser 488 nm; red laser 638 nm) was fixed (final concentration 0.5% glutaraldehyde, Sigma Aldrich, MA, USA; for SAS samples for eukaryotic cell enumeration including 0.01% Pluronic acid F-68, Gibco, MA, USA), incubated at room temperature for 5 min and frozen at -80°C.
Instrument	CYTOFlex, Beckman Coulter Inc, CA, USA; blue laser 488 nm; red laser 638 nm
Software	CytExpert
Cell population abundance	No sorting was performed. See Table 1 for abundances of quantified groups.
Gating strategy	Bacteria were enumerated after staining with SYBR Green I (0.06% final concentration, Invitrogen, MA, USA) with detection of microbial cells using the blue laser (488 nm) and a combination of side scatter and green fluorescence at 525/40 nm. To quantify nanophytoplankton and photosynthetic picoeukaryotes (PPE), 488 nm forward scatter was used as a proxy for cell diameter and red fluorescence at 690/50 nm as a proxy for chlorophyll a.

- [ ] Tick this box to confirm that a figure exemplifying the gating strategy is provided in the Supplementary Information.
